# CFD Investigation of Spacer-Filled Channels for Membrane Distillation

**DOI:** 10.3390/membranes9080091

**Published:** 2019-07-25

**Authors:** Mariagiorgia La Cerva, Andrea Cipollina, Michele Ciofalo, Mohammed Albeirutty, Nedim Turkmen, Salah Bouguecha, Giorgio Micale

**Affiliations:** 1 Dipartimento di Ingegneria, Università degli Studi di Palermo, viale delle Scienze ed.6, 90128 Palermo, Italy; 2 Center of Excellence in Desalination Technology, King Abdulaziz University, Jeddah 21589, Saudi Arabia; 3 Mechanical Engineering Department, King Abdulaziz University, Jeddah 21589, Saudi Arabia

**Keywords:** desalination, membrane distillation, spacer-filled channel, temperature polarization, computational fluid dynamics, thermochromic liquid crystals

## Abstract

The membrane distillation (MD) process for water desalination is affected by temperature polarization, which reduces the driving force and the efficiency of the process. To counteract this phenomenon, spacer-filled channels are used, which enhance mixing and heat transfer but also cause higher pressure drops. Therefore, in the design of MD modules, the choice of the spacer is crucial for process efficiency. In the present work, different overlapped spacers are investigated by computational fluid dynamics (CFD) and results are compared with experiments carried out with thermochromic liquid crystals (TLC). Results are reported for different flow attack angles and for Reynolds numbers (Re) ranging from ~200 to ~800. A good qualitative agreement between simulations and experiments can be observed for the areal distribution of the normalized heat transfer coefficient. Trends of the average heat transfer coefficient are reported as functions of Re for the geometries investigated, thus providing the basis for CFD-based correlations to be used in higher-scale process models.

## 1. Introduction

In regions where the existing freshwater sources do not meet the water demand, production of potable water from saline and brackish waters is needed [1]. Among the desalination processes, membrane distillation (MD) has recently attracted significant interest due to its features, which make MD a process easily scalable to small–medium size [2,3,4]. MD is a thermally driven membrane-based process where the transport of water vapor is realized through a micro-porous hydrophobic membrane, which leads to a theoretical 100% rejection of salts. The separation process is driven by the vapor pressure difference at the membrane/liquid interfaces due to the temperature gradient existing between the two sides of the membrane. MD is characterized by its compactness and low operational pressure requirement as well as low working temperature. These features make it possible to power MD by low grade heat (waste heat) or renewable energy and boost its applicability in remote areas and small scale production [5,6,7]. Another important distinct feature of MD is its ability to desalinate highly saline brines and wastewater. MD is the most appropriate option to increase the recovery ratio of multi-stage flash (MSF) and reverse osmosis (RO) plants due to it its ability to treat feeds with high salinity and impurities. One main advantage of MD technology is that evaporation and condensation surfaces are tightly packed, thus leading to a compact system with low capital cost per unit product. The classification of MD systems is related to the adopted condensation methods. In general, MD systems are classified into four basic simple configurations designed relative to the permeate vapor condensation method used. These include direct contact membrane distillation (DCMD), air gap membrane distillation (AGMD), sweeping gas membrane distillation (SGMD), and vacuum membrane distillation (VMD) [5]. The succession of these different configurations was dictated by a need to improve the performance of the process. DCMD is the simplest configuration that has liquid phases (hot feed and cold permeate) in direct contact with both sides of the porous hydrophobic membrane. The vapor diffusion path is limited to the thickness of the membrane, therefore reducing the mass and heat transfer resistances and thus enhancing the process [8,9].

Despite these advantages and the many experimental and theoretical studies conducted in recent years, MD is not yet fully developed from the commercial point of view because it is still highly energy demanding (typical thermal energy consumption ~100 kWh/m^3^) compared, for example, to multiple effect distillation, or MED (thermal energy consumption ~40 kWh/m^3^ for large plants) and reverse osmosis, or RO (mechanical energy consumption 2–4 kWh/m^3^) [10,11,12]. Other reasons why MD is still on a pilot scale are related to membrane, module design, and low water flux. One of the main causes responsible for low water flux is the temperature polarization phenomenon, while the concentration polarization has a negligible effect on the permeate flow rate reduction [13]. Temperature polarization can lead to a dramatic reduction of the actual transmembrane temperature driving force [10,13]. A reduction of these effects and a performance improvement could be achieved by the optimization of the geometry of the channels and of the adopted spacers acting as mixing promoters.

Computational fluid dynamics (CFD) is suitable to investigate flow field and transport phenomena in spacer-filled channels. Since the 1990s, CFD has been used to study channels with spacers, by 2D [14,15,16] or 3D simulations [17,18,19,20], in the steady regime [16,21,22,23,24] or considering unsteady flows [25,26,27]. In the unsteady regime, very accurate direct numerical simulations (DNS) become computationally expensive, and turbulence models may be more suitable [28,29]. In regard to CFD results validation, in most of the literature only global quantities from CFD and experiments are compared, such as the friction coefficient and the average Nusselt or Sherwood numbers [25,30,31]. However, local data (i.e., flow fields and temperature distributions) are more suitable than global quantities because they better allow one to check if the assumptions made in CFD simulations (e.g., assuming simple boundary conditions or neglecting geometrical irregularities) reproduce sufficiently well the actual physics of the system. Experiments based on thermochromic liquid crystals (TLC) and digital image processing (DIP) can provide local results, such as temperature and heat transfer coefficient distributions, which can also be processed to obtain global quantities [27,32,33,34,35].

The aim of the present work is to characterize and design novel prototypes of spacers for membrane separation modules. We performed 3D simulations from the laminar to the unsteady regime by using, in this latter case, the shear stress transport (SST) turbulence model. For selected test cases, CFD results were compared with experimental data obtained in a purpose built test facility where the TLC technique was used. The validated CFD model was then used to investigate several spacers in different conditions.

## 2. Materials and Methods

### 2.1. Geometry, Operating Conditions, and Performance Parameters

**Figure 1:**
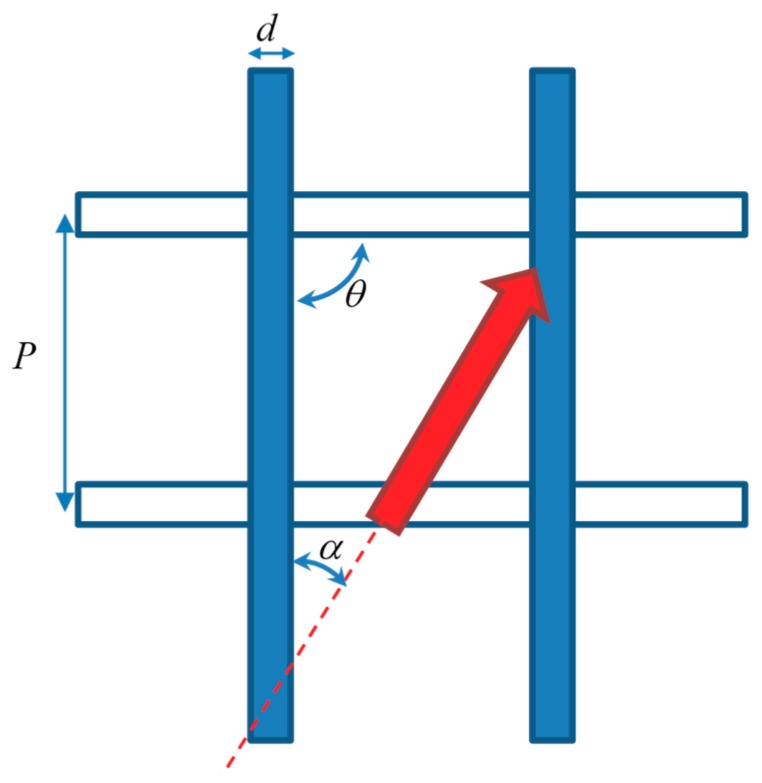
Sketch of a generic overlapped spacer with the main geometric parameters. The red arrow indicates the flow direction.

An overlapped spacer of the kind examined in the present study is characterized by a few geometric parameters (Figure 1), namely: the filament diameter (*d*); the orthogonal distance between filaments (pitch, *P*); the intrinsic angle between filaments (*θ*); and the flow attack angle (*α*) between the directions of the main flow and of the filament layer adjacent to a specified (e.g., top) wall. Note that *d* and *P* are assumed to be the same for the two overlapped layers of filaments. In the present study, for the above parameters the values reported in Table 1 were investigated by both experiments and CFD simulations. In order to exactly reproduce the geometry of the experimental module, the geometrical model for CFD was created from the same CAD files used for the 3D printing of the spacer. The channel height *H*, chosen to be within the range of typical spacer thickness values in MD [18,36,37], was not exactly equal to 2*d* (twice the filament diameter), i.e., 4 mm, but slightly less (3.8 mm) because a certain amount of interpenetration existed. Thus, the pitch to channel height ratio was *P*/*H* = 2.63.

Both in the experiments and in the computations, the bottom wall was assumed adiabatic and heat transfer occurring only through the top one (active wall), in which distributions of temperature, wall heat flux, and heat transfer coefficient were assessed. This last quantity was locally defined as:

(1) $$h=\frac{q^{\prime \prime }}{T_{bulk}-T_{wall}}$$

in which *T_bulk_* is the local fluid bulk temperature, while *q*″ and *T_wall_* are the local wall heat flux and wall temperature, respectively. For the reasons discussed in [23], an average heat transfer coefficient is better defined not as the surface average 〈*h*〉 of the local *h*, but rather as

(2) $$h_{avg}=\frac{\langle q^{\prime \prime }\rangle }{T_{bulk}-\langle T_{wall}\rangle }$$

in which the symbol 〈 〉 denotes averaging operation on the active wall.

The Reynolds number (Re) is defined as:

(3) Re=UDhν

where *D_h_* is the hydraulic diameter, conventionally assumed to be equal to twice the channel height (7.6 mm) as in a void plane channel of infinite width. *U* is the approach, or superficial, velocity, defined as the velocity that the fluid would have if the channel were void of any spacer; it is equal to the ratio between the flow rate and the passage area *U* = *Q*/*A_p_* (in the experiments *A_p_* = 9.12 × 10^−4^ m^2^). ν is the kinematic viscosity, which was assumed equal to 6.78 × 10^−7^ m^2^/s in the cases investigated (water at ~43 °C).

For each configuration, the values of the flow rate investigated and the corresponding values of the Reynolds number are those given in Table 2. Flow rates were selected based on the experiments’ limitations (the range of the flowmeters, measurement uncertainty at low flow rates, and pressure build up at the Plexiglas channel), and to enable comparison of results with several studies conducted in the literature by other researchers within the same range of values.

### 2.2. Experimental Set-Up and Procedures

**Figure 2:**
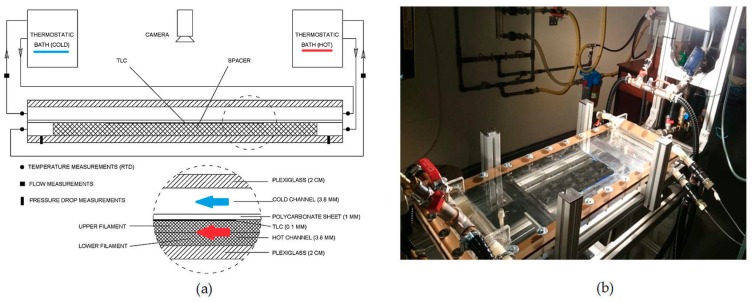
(**a**) Schematic representation and (**b**) picture of the experimental setup.

The experimental results were obtained in a previous study [35] using the setup shown in Figure 2. It is composed of two narrow (~4 mm height) hot and cold water channels separated by a 1 mm thick polycarbonate sheet. These channels are enclosed by an upper and a lower 2 cm thick transparent Plexiglass sheet. Test spacers were custom designed and fabricated using 3D printing with overall dimensions of 24 cm × 24 cm × 0.38 cm. The spacers were designed to fit and fill the hot channel while touching a thermochromic liquid crystals (TLC) sheet, which was made to adhere to the lower surface of the polycarbonate sheet. TLC sheets adopted had a color play from 35 to 40 °C. The hot and cold water temperatures were measured using RTD sensors located at the inlet and outlet of the two channels, while the flow rates were measured using flow sensors and rotameters. Julabo temperature-controlled heating and cooling circulators were used to control the hot and cold water temperatures at the channel inlets. Further details on experimental procedure and data processing can be found in [35]. The color distribution of the TLC at the upper surface of the hot channel was monitored by a high resolution camera, and images (1920 × 1080 pixels) were captured for different feed temperatures and flow rates (from 1 to 4 L/min), while the flow rate in the cold channel was kept at 8 L/min.

These images were processed by first converting them to the HSV (hue, saturation, value) format. It is known that the hue component of this format correlates with temperature but requires prior calibration. At the beginning of each spacer experiment, a TLC calibration test was preliminarily performed and a best fit polynomial curve was obtained for the temperature as a function of hue. During the calibration test both the upper and lower channels were fed by the same hot water source. In the real experiments, the cold water was set to 30 °C and the hot water was set to 43 °C, allowing a 13 °C difference between the two channels for heat transfer. Hot and cold water inlet temperatures were selected after several pre-tests to ensure that the temperature distributions on the TCL surface were clearly visible and fell within the color play range for all the flow rates.

It should be observed that in order to characterize the temperature polarization phenomenon, it is sufficient to reproduce the thermal field and convective heat transfer occurring in the spacer-filled channel only. This can be achieved by imposing at the active wall a realistic thermal boundary condition, representative of those occurring in MD modules, independent of whether there are or not an actual membrane and a vapor flux.

### 2.3. CFD Investigations

The problem was described by the continuity, momentum (Navier–Stokes), and energy equations for water at *T* = 43 °C and *p* = 1 bar, which were solved by the ANSYS-CFX^®^ code (ANSYS, USA). In the simulations, the unit periodic cell approach was used [22,34,38], which simulates flow and heat exchange phenomena in periodic lattices under fully developed conditions (i.e., at sufficient distance from inlets and outlets). A source term, accounting for the large-scale temperature gradient, was implemented in the energy equation, and a body force per unit volume, accounting for the large-scale pressure gradient, in the momentum equations. In each run, this latter term was dynamically adjusted to obtain the required Re value (corresponding to one of those achieved in the experiments).

As reported in the literature, for geometries similar to those examined here [19,26], the fluid flow becomes unsteady for Re > 350. For this reason, steady-state laminar simulations were carried out for Re = 205 and 305, while, for 410 ≤ Re ≤ 820, the shear stress transport (SST) turbulence model was used, which is a blend between the k-ω model near the walls and the k-ε model in the outer region. As demonstrated in previous works [29], for the present transitional flows, ω-based models, which fully resolve the near-wall layer, are preferable to ε-based models, which make use of wall functions; among them, the SST model gives the most accurate predictions in terms of both distributions and average values of the heat transfer coefficient.

**Figure 3:**
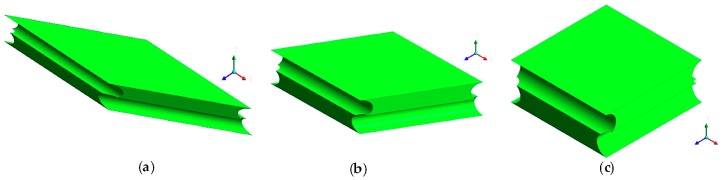
Computational domain (unit cell): (**a**) *θ* = 30°; (**b**) *θ* = 60°; (**c**) *θ* = 90°.

**Figure 4:**
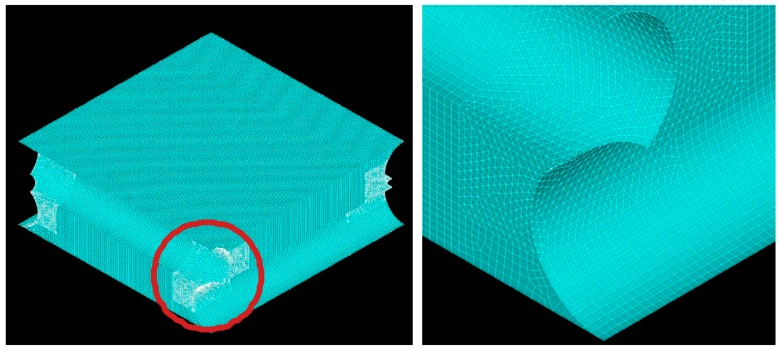
Mesh of a unit cell for case (**c**); one of the regions meshed with tetrahedra is highlighted.

The computational domains (different according to the intrinsic angle) are shown in Figure 3, while details of the computational finite volume grid for case (c) are shown in Figure 4. Only four corner blocks were meshed with tetrahedra; the remaining volume was meshed with mapped hexahedra or with sweep mode in the regions adjacent to the cylindrical surfaces. In regard to the boundary conditions, periodicity was imposed at the walls perpendicular to the filaments; at the other walls a no slip condition was set. The fluid–filament interfaces and the bottom wall were assumed adiabatic, while at the top wall a mixed condition (Robin) was imposed by prescribing an external temperature of 30 °C and an external heat transfer coefficient equal to λ/δ, λ being the fluid’s thermal conductivity and δ the channel thickness.

## 3. Results and Discussion

### 3.1. CFD Prediction of Temperature and Heat Transfer Coefficient Distributions and Comparison with Experiments

**Figure 5:**
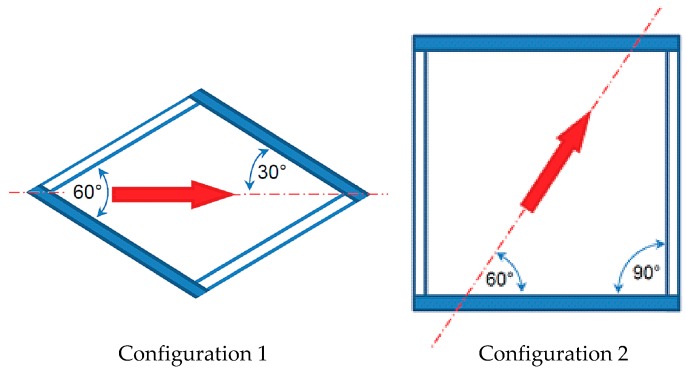
Sketch of the configurations for which experimental and computational distributions of the normalized heat transfer coefficient are compared. The intrinsic angle *θ* between the filaments and the flow attack angle *α* are reported.

For the sake of brevity, results are illustrated here in detail only for the two geometrical configurations in Figure 5. Configuration 1 is characterized by an intrinsic angle *θ* between filaments of 60° and a flow attack angle *α* of 30°, while configuration 2 is characterized by *θ* = 90° and *α* = 60°. The solid profiles indicate the spacer filaments, which touch the upper, thermally active, wall. The results of the whole computational campaign (aiming at the determination of the overall dependence of 〈*h*〉 on Re for each geometrical configuration, to be used in process modelling applications) are reported in the final section.

For each configuration, three Reynolds numbers were considered: the lowest (205), an intermediate one (410), and the highest (820) of those reported in Table 2. Distributions of *h*, normalized by the mean value 〈*h*〉, are shown. Experimental distributions were obtained by post-processing the TLC images, as described in Section 2.2, and averaging the results over 3 × 3 unit cells to reduce dispersion.

#### 3.1.1. Configuration 1

**Figure 6:**
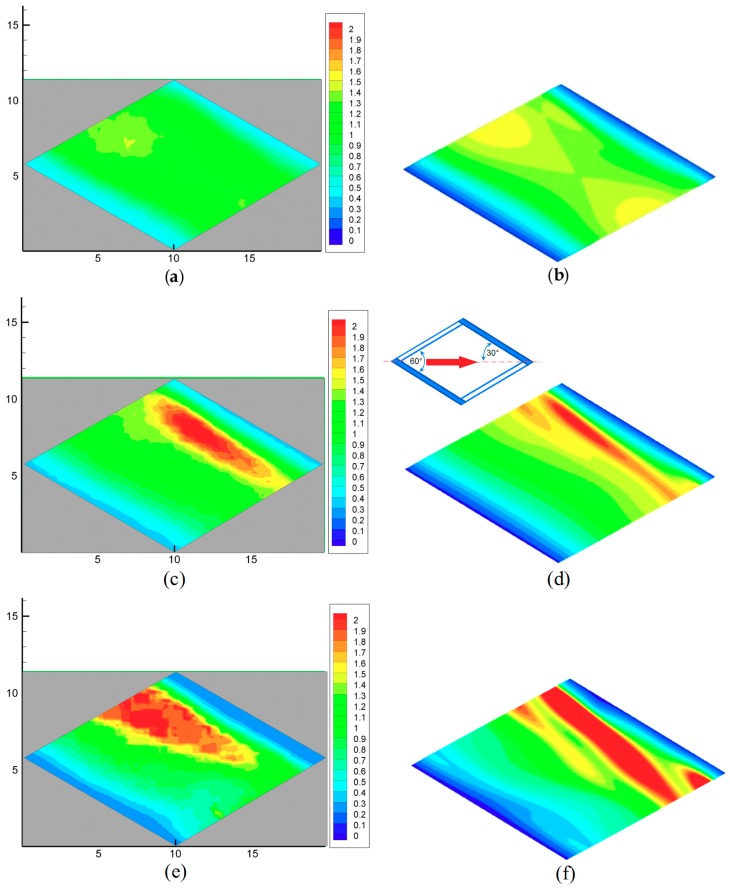
Configuration 1: distributions of the normalized heat transfer coefficient for increasing flow rates. (**a**,**b**) *Q* = 1 L/min. Re ≈ 205; (**c**,**d**) *Q* = 2 L/min. Re ≈ 410; (**e**,**f**) *Q* = 4 L/min. Re ≈ 820. Left column (**a**,**c**,**e**) experimental results; right column (**b**,**d**,**f**) computational fluid dynamics (CFD) predictions. Coordinates are in mm.

Results for configuration 1 are reported in Figure 6. For clarity purposes, the insets show the direction of the flow and the arrangement of the filaments.

The comparison shows a fair overall agreement between simulations and experiments in the heat transfer coefficient distribution. At the lowest Re, the *h* distribution was roughly symmetric between the upstream (left) and downstream (right) halves of the cell, showing that inertial effects were negligible. As Re increased, a stripe of low *h* values grew shortly after the upstream upper filament, associated with a region of separated flow (as confirmed by an analysis of the CFD-predicted flow fields). At the same time, a band of high *h* values appeared shortly before the downstream upper filament, in correspondence with the reattachment of the separated shear layer. Some discrepancy existed between experiments and CFD results, which predicted stripes of high *h*, narrower, longer, and inclined with respect to the filaments. Note also that experimental results showed residual fluctuations, not eliminated by the spatial averaging performed (which only involved nine unit cells); CFD simulations, by their nature themselves, yielded time-averaged values of *h* (and of any other variable) and thus appeared smooth and regular.

#### 3.1.2. Configuration 2

**Figure 7:**
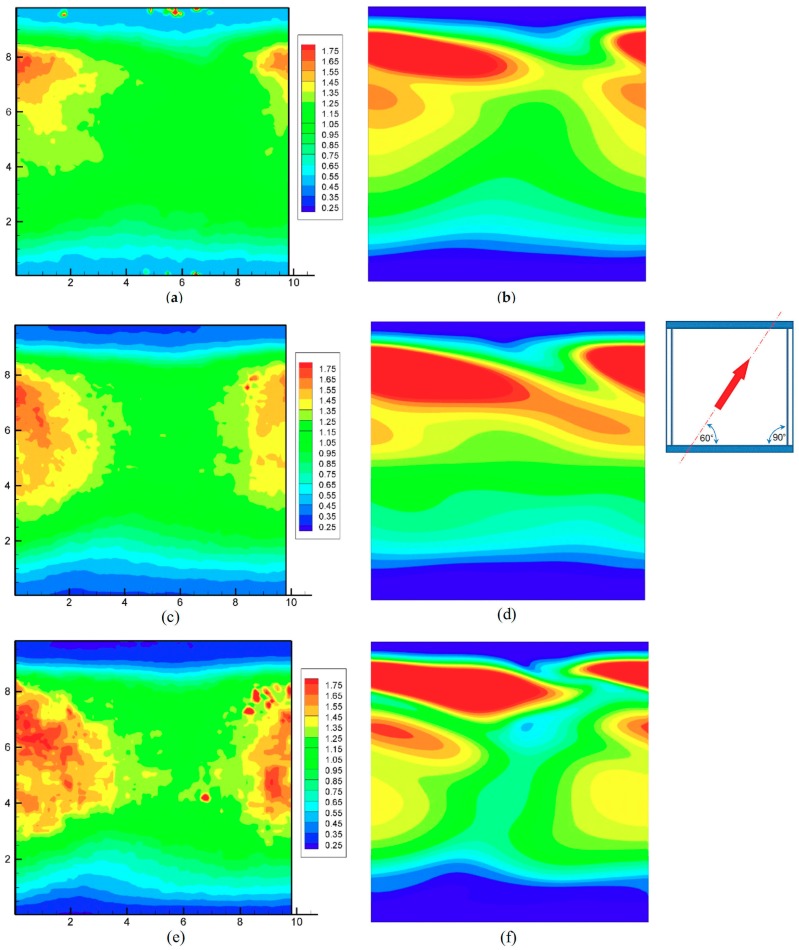
Configuration 2: distributions of the normalized heat transfer coefficient for increasing flow rates. (**a**,**b**) *Q* = 1 L/min. Re ≈ 205; (**c**,**d**) *Q* = 2 L/min. Re ≈ 410; (**e**,**f**) *Q* = 4 L/min. Re ≈ 820. Left column (**a**,**c**,**e**) experimental results; right column (**b**,**d**,**f**) CFD predictions. Coordinates are in mm.

Results for configuration 2 are reported in Figure 7. As in the previous Figure 6, the inset shows the direction of the flow and the arrangement of the filaments.

In this case only a partial agreement between simulation and experiments could be registered. The numerical simulations predicted, both under laminar flow conditions (Figure 7b) and using the turbulence model (Figure 7d,f), a band where the most intense heat transfer occurs, located shortly before the downstream (upper) spacer filament, and a secondary band located shortly upstream of it (i.e., closer to the center of the unit cell). These features were not present in the experimental results, which showed a broad maximum of *h* only slightly upstream of the upper filament and centered on the opposite filament (the one that did not touch the active upper wall).

General levels and the overall distribution of *h*/*h_avg_*, however, were satisfactorily reproduced. As in the previous configuration, experimental results exhibited a considerable amount of residual fluctuations, not completely damped by the 9-cell spatial averaging performed, while CFD results were smooth and regular.

### 3.2. CFD Prediction of Average Heat Transfer Coefficient and Comparison with Experiments

**Figure 8:**
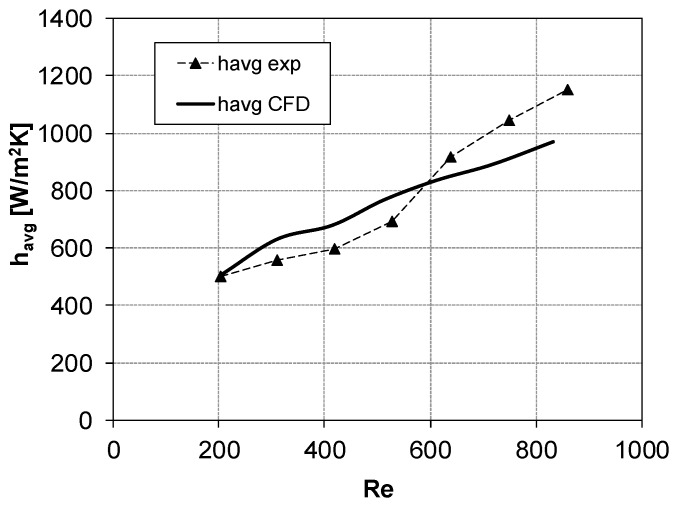
*h_avg_* vs. Re for *θ* = 60°, *α* = 30° (configuration 1). Symbols denote the experimental results, while CFD results are represented by the thick solid line.

**Figure 9:**
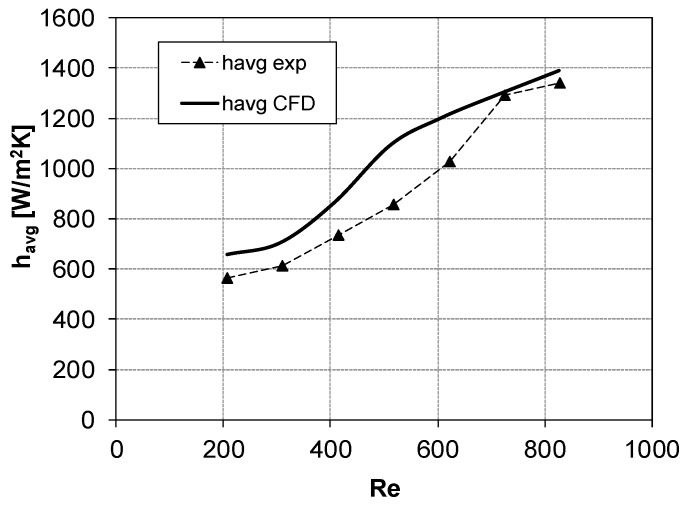
*h_avg_* vs. Re for *θ* = 90°, *α* = 60° (configuration 2). Symbols denote the experimental results, while CFD results are represented by the thick solid line.

Figure 8 and Figure 9 show the average heat transfer coefficient *h_avg_* (Equation (2)) as a function of the Reynolds number for configurations 1 (*θ* = 60°, *α* = 30°) and 2 (*θ* = 90°, *α* = 60°), respectively.

A fair agreement between the experiments and the CFD simulations could be noticed. CFD results for configuration 1 (Figure 8) overpredicted *h_avg_* by ~13% when Re was lower than ~600; at higher Re, a slight underprediction was registered (~14%). In Figure 9, results for configuration 2 showed that CFD overestimates *h_avg_* by ~14%, with the maximum difference between experiments and simulations (~25% overprediction) observed at Re ≈ 500–600.

### 3.3. Summary Results for All Geometries Investigated

**Figure 10:**
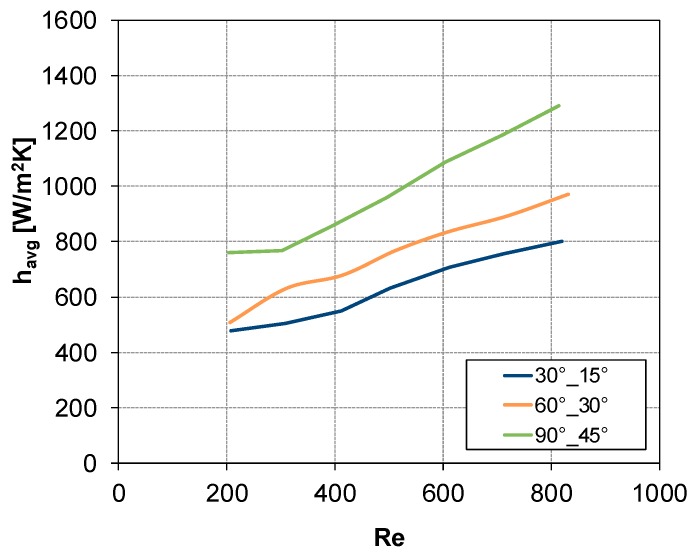
CFD results: *h_avg_* vs. Re for *θ* = 30°,*α* = 15°; *θ* = 60°, *α* = 30°; and *θ* = 90°, *α* = 45°.

**Figure 11:**
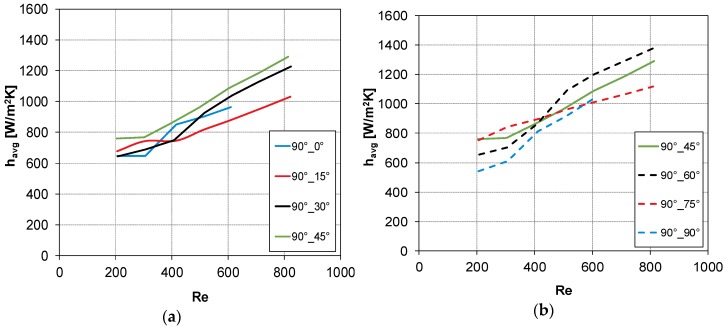
CFD results: *h_avg_* vs. Re for *θ* = 90°: (**a**) *α* ≤ 45° and (**b**) *α* ≥ 45°.

In Figure 10 and Figure 11, the CFD results obtained for all the configurations reported in Table 1 are shown. In Figure 10, *h_avg_* is plotted as a function of Reynolds number for the three configurations in which the flow direction bisects the intrinsic angle *θ*, i.e., *θ* = 30°, *α* = 15°; *θ* = 60°, *α* = 30°; and *θ* = 90°, *α* = 45°. Curves of *h_avg_* against Re, for the configuration with *θ* = 90°, are reported in Figure 11 considering flow attack angles values lower (graph a) or higher (graph b) than 45°.

From Figure 10 and Figure 11, it is possible to identify the configuration with *θ* = 90°, *α* = 60° as that providing the highest heat transfer coefficient.

## 4. Conclusions

Flow and heat transfer were predicted by computational fluid dynamics for channels provided with spacers consisting of two overlapped layers with a fixed pitch to height ratio (~2.63) and different values of the intrinsic angle between the filaments and of the flow attack angle. The Reynolds number ranged between 200 and 820.

In a previous work, experiments were carried out for the same configurations and Reynolds numbers by using thermochromic liquid crystals (TLC), which provide the distribution of the heat transfer coefficient *h* on the active wall.

For two selected cases, distributions of *h* obtained by CFD were compared with the experimental distributions, obtained by post-processing the real images of the TLC. From the comparison, a fair agreement concerning the shape of the *h* distributions was registered; the match was better at low Reynolds number, when no turbulence model was used, and worse at high Re, when simulations relied on the SST turbulence model.

For the same test cases, the average heat transfer coefficient was also fairly well predicted by CFD simulations, with discrepancies of the order of 10–20%, which can be regarded as only minor in view of the complex geometry and flow structure.

Finally, the results of the whole computational campaign were reported in terms of average heat transfer coefficient as a function of the Reynolds number and can be used in process modelling applications involving spacers with the same geometrical features as those investigated here.

In MD systems, geometrical configurations yielding the highest possible heat transfer coefficient *h* should be adopted in order to minimize the membrane surface (the largest component of plant cost). With reference to the geometries investigated in this work, for Reynolds numbers above 400–500, this optimal configuration seems to be characterized by an intrinsic angle *θ* of 90° and a flow attack angle *α* of 60°.

## Figures and Tables

**Table 1:** Investigated values of geometric parameters of spacers.

*d* (mm)	*P* (mm)	*θ* (°)	*α* (°)
2	10	30	15
		60	30
		90	0
			15
			30
			45
			60
			75
			90

**Table 2:** Investigated values of the flow rate and corresponding Reynolds numbers (Re).

*Q* (L/min)	Re
1	205
1.5	307
2	410
2.5	512
3	615
3.5	717
4	820
